# Impact of Gastric Acid Suppression on Clostridioides difficile Infection: A Systematic Review

**DOI:** 10.7759/cureus.115565

**Published:** 2026-08-31

**Authors:** Ajay Kausheic Madhusuthanan, Maryam Afzal, Diana Turuzhbaeva, Hala Rahman, Mithum Senaratne, Mona Ajal Loueian, Rahul Goyal, Summayya Anwar

**Affiliations:** 1 Emergency Medicine, Apollo Hospitals, Chennai, IND; 2 Dentistry, Primary and Secondary Healthcare, Rawalpindi, PAK; 3 Internal Medicine, Samara State Medical University, Samara, RUS; 4 Trauma and Orthopaedic Surgery, Midland Regional Hospital of Tullamore, Tullamore, IRL; 5 Surgery, Faculty of Medicine University of Ruhuna, Galle, LKA; 6 Medicine, American University of Integrative Sciences, Saint Michael, BRB; 7 Trauma and Orthopaedics, King's Mill Hospital, Sutton-in-Ashfield, GBR; 8 Biosciences, COMSATS University Islamabad, Islamabad, PAK; 9 Research and Development, California Institute of Behavioral Neurosciences and Psychology, Fairfield, USA

**Keywords:** acid-suppressive therapy, cdi risk, clostridioides difficile infection, gastric acid suppression, h2 receptor antagonists, ppis, proton pump inhibitors

## Abstract

*Clostridioides difficile* infection (CDI) is a leading cause of healthcare-associated infections, posing significant morbidity, mortality, and financial burden. While antibiotic use remains the primary risk factor, increasing attention has been given to the role of gastric acid suppression-particularly proton pump inhibitors (PPIs) and histamine-2 receptor antagonists (H₂RAs)-in facilitating CDI. Gastric acid acts as a natural barrier against ingested pathogens; suppression of this barrier may allow *C. difficile* spores to survive and colonize the gut. The aim of this article is to systematically review and evaluate existing evidence on the association between gastric acid suppression and the risk of developing CDI, and to explore the potential dose-response relationship and implications for clinical practice. A comprehensive search was conducted using PubMed, Google Scholar, and MEDLINE for studies published between 2007 and 2025. Inclusion criteria focused on human studies evaluating the relationship between acid-suppressive therapy and CDI risk. Studies were appraised using validated tools such as the Critical Appraisal Skills Programme (CASP), A MeaSurement Tool to Assess systematic Reviews 2 (AMSTAR 2), and the Scale for the Assessment of Narrative Review Articles (SANRA). Six studies met the inclusion criteria: two retrospective cohort studies, one meta-analysis, one systematic review, one narrative review, and one case series. Most studies consistently found an association between PPI use and increased CDI risk, including pediatric populations. A dose- and duration-dependent relationship was reported, with longer or higher exposure to PPIs correlating with greater CDI risk. The meta-analysis and systematic review provided the strongest evidence, reinforcing causality and the need for cautious prescribing. Notably, PPIs were often continued even after CDI diagnosis, highlighting gaps in deprescribing practices. There is compelling observational evidence linking PPIs-and, to a lesser extent, H₂RAs-with an elevated risk of CDI. This risk spans age groups and appears to intensify with prolonged use. While randomized controlled trials are needed to confirm causality, current evidence supports more judicious use of acid-suppressive therapy, regular medication reviews, and the incorporation of deprescribing strategies to reduce CDI incidence.

## Introduction and background

*Clostridioides difficile* infection (CDI) remains one of the leading causes of healthcare-associated infections worldwide and is associated with substantial morbidity, mortality, and healthcare costs [1]. Although antibiotic exposure is the most well-established risk factor for CDI, increasing evidence suggests that additional contributors-including gastric acid suppression therapy-may also play a significant role in both hospital-acquired and community-acquired infections [2].

Gastric acid serves as an essential first-line defense against ingested pathogens. The stomach normally maintains a highly acidic environment (pH 1-2), which limits bacterial survival and reduces microbial colonization of the upper gastrointestinal tract [3]. Suppression of gastric acid secretion through proton pump inhibitors (PPIs) or histamine-2 receptor antagonists (H₂RAs) may compromise this protective barrier, facilitating the survival and intestinal transit of *C. difficile* spores [4]. Beyond impaired gastric defense, acid suppression has also been implicated in alterations of gut microbiota composition, including reduced microbial diversity and increased susceptibility to colonization by opportunistic pathogens [4].

Multiple observational studies and pooled analyses have demonstrated a significant association between PPI use and an increased risk of incident CDI [5-7]. Some studies have further suggested a dose-response relationship, strengthening the possibility of a causal association [8]. Although H₂RAs appear to confer a comparatively lower risk than PPIs, several analyses have still identified a statistically significant association between H₂RA exposure and CDI [9]. Several meta-analyses have shown that continued PPI use during or after CDI treatment increases the likelihood of recurrence [10]. Subsequent epidemiological studies and meta-analyses have consistently supported these observations across diverse clinical populations [11-18]. Large population-based studies have reinforced these findings in both community and hospital settings. For example, Dial et al. first reported an association between acid-suppressive therapy and community-acquired CDI [19], while Howell et al. demonstrated increased risk among hospitalized patients [20]. PPI use has been identified as an independent risk factor for healthcare-associated diarrhea, underscoring its potential contribution to the development of CDI in hospitalized patients [21]. Several large meta-analyses have confirmed that PPI exposure is associated with a significantly increased risk of both community-acquired and healthcare-associated CDI [22-25].

Continued PPI therapy has been associated with an increased risk of recurrent CDI, suggesting that persistent acid suppression may hinder restoration of normal gut microbiota after initial treatment [26]. In addition to primary infection, acid suppression has also been linked to recurrent CDI. The proposed mechanisms include persistent dysbiosis, impaired restoration of normal intestinal microbiota, and diminished mucosal immune responses [4,27].

Despite the consistency of epidemiological data, most available studies are observational and therefore susceptible to confounding factors such as concomitant antibiotic exposure, co-morbid illnesses, and healthcare contact [2,28]. Nevertheless, the presence of dose-response relationships, reproducibility across settings, and supportive mechanistic evidence collectively strengthen the hypothesis that gastric acid suppression may contribute-at least in part-to the pathogenesis of CDI [28-30]. Experimental and microbiome studies further support the biological plausibility of these associations by demonstrating that PPIs significantly alter gut microbial composition in ways that favor pathogen overgrowth [27,29,30].

The present systematic review aims to critically evaluate the current body of evidence regarding the impact of gastric acid suppression induced by PPIs and H₂RAs on the risk of CDI development and recurrence. By integrating epidemiological findings with mechanistic insights, this review seeks to clarify the potential role of acid-suppressive therapy in CDI and to inform future clinical decision-making.

## Review

Screening and selection process

All titles and abstracts were initially screened independently by co-authors. Relevant studies then underwent full-text review to confirm eligibility. Duplicate articles were removed prior to screening. Any discrepancies between reviewers were resolved through discussion or with the help of a third reviewer.

Statistical analysis and data synthesis

Because the included studies differed substantially in study design, population, exposure definitions, outcome assessment, and reported measures of association, a new quantitative meta-analysis was not performed. The findings were therefore synthesized narratively. Where reported in the original studies, measures of association such as odds ratios, risk ratios, and corresponding 95% confidence intervals and P-values were extracted and summarized. The results of previously published meta-analyses were reported as presented in the original publications. Differences in study characteristics and methodological quality were considered when interpreting the consistency and strength of the evidence.

Inclusion and exclusion criteria

Studies were included if they met the following criteria: studies involving gastric acid suppressants and reporting on outcomes such as CDI, gut microbiota changes, or altered gastrointestinal pH. Only studies published in English and available in full text were included. Studies were excluded if they were animal or in vitro studies, involved only pediatric populations, or were non-English, duplicate, or non-peer-reviewed (Table 1).

**Table 1:** Articles identified using each database

Databases	Keywords	Search strategy	Filters	Search result
PubMed	Antacids and *C. difficile*	( "Antacids/administration and dosage"[Mesh] OR "Antacids/adverse effects"[Mesh] OR "Antacids/pharmacology"[Mesh] ) AND ( "Clostridioides difficile/cytology"[Mesh] OR "Clostridioides difficile/drug effects"[Mesh] OR "Clostridioides difficile/metabolism"[Mesh] OR "Clostridioides difficile/pathogenicity"[Mesh] )	English, human, 2007-2025	3
PubMed	Antacids and *C. difficile*		English, human, 2007-2025	29
PubMed Advanced	Antacids and *C. difficile*	((clostridiodes difficile[Title/Abstract]) AND (antacids[Title/Abstract])) AND (("2007"[Date - Publication] : "3000"[Date - Publication]))	English, human, 2007-2025	5
Google Scholar	Antacids and *C. difficile*	Antacids and *Clostridioides*	2007-2025	588
MEDLINE	Antacids and *C. difficile*	Antacids and *Clostridioides difficile*		5
BMC Medicine	Antacids and *C. difficile*	*C. difficile* and antacids		2
PubMed Central		*C. difficile* and antacids	Last 10 years	161

Of all the studies that met the above criteria, further scrutiny was done, and articles that passed the scrutiny were accepted. Figure 1 shows the PRISMA flowchart of the number of articles identified from each database and how many were retrieved after quality appraisal.

**Figure 1:**
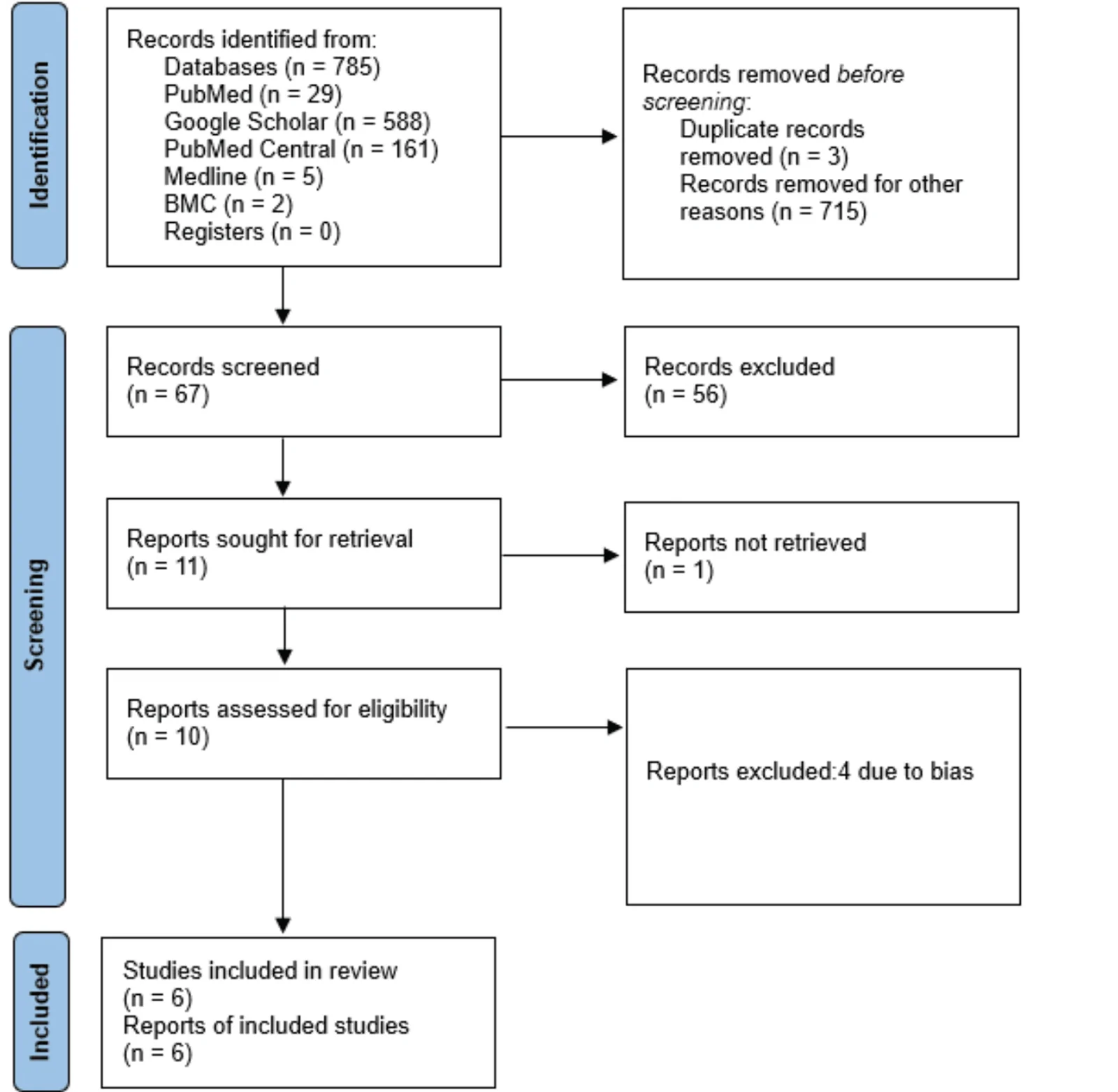
PRISMA flow diagram

Quality appraisal and process of collecting data

The appropriate quality appraisal tools were applied to the nominated studies according to their type of study. We used the Critical Appraisal Skills Programme (CASP) checklist to assess the cohort studies, the Scale for the Assessment of Narrative Review Articles (SANRA) to assess the narrative reviews, and A MeaSurement Tool to Assess systematic Reviews (AMSTAR) for meta-analyses. The co-authors assessed the quality of the studies independently. After collecting all the relevant papers, each co-author contributed to the evaluation data for that systematic review. Criteria used for quality appraisal are shown in Table 2.

**Table 2:** Quality assessment of included studies ★: criterion not or poorly met; ★★: criterion partially met; ★★★: criterion fully met; *: not applicable; PPI: proton pump inhibitor; H₂RA: histamine-2 receptor antagonist; CDI: *Clostridioides difficile* infection; OR: odds ratio; RR: risk ratio; CI: confidence interval

Quality criterion	Linsky et al. (2010) [11]	Mezoff and Cohen (2013) [12]	Finke et al. (2025) [15]	Głaz et al. (2023) [16]	Okamoto et al. (2023) [17]	Huang et al. (2024) [18]
Clear research question/objective	★★★	★★★	★★★	★★★	★★★	★★★
Inclusion & exclusion criteria clearly defined	★★★	★★★	★★	★★★	★★	★★★
Valid exposure measurement (PPI/H_2_RA use)	★★	★★	★★★	*	★★★	★★★
Reliable outcome definition for CDI	★★★	★★	★★★	*	★★	★★★
Adequate sample size	★★★	★★★	★★★	*	★★	★★★
Adjustment for confounders	★★	★★	★★★	*	★★	★★
Assessment of dose/duration	★★	★★	★★★	*	★	★★★
Reporting of effect estimates (OR, RR, CI)	★★★	★★★	★★★	*	★★	★★★
Risk of bias assessment reported	★★	★★★	★★★	★★★	★	★★
Appropriateness of study design	★★★	★★★	★★★	★★★	★★★	★★★
Transparency of methodology	★★★	★★★	★★★	★★	★★	★★★

Most of the studies were generally good in quality (Table 2), with clear research questions and suitable study designs. Some studies measured PPI use and CDI outcomes very well, while others were less clear. Sample sizes were mostly adequate, but not all studies properly adjusted for confounders or looked at dose and duration of PPI use. Most studies reported effect estimates clearly. A few studies also had weaker methods or did not assess bias fully. Overall, the research is useful, but some studies are stronger than others, so the findings should be interpreted with care.

The studies reviewed included a mix of cohort studies, observational work, systematic reviews, meta-analyses, and a narrative review mentioned in Table 3. Most were assessed using standard appraisal tools such as CASP, AMSTAR, or SANRA and showed low to moderate risk of bias. Across these studies, the main aim was to explore different aspects of acid suppression therapy and its potential relationship with CDI-ranging from overall risk, dose-response patterns, deprescribing practices, and duration of use. This systematic review consolidates the available evidence on the relationship between gastric acid suppression and the risk of CDI. Across six included studies encompassing retrospective cohorts, meta-analyses, systematic reviews, narrative synthesis, and practice-based observational designs, a consistent association was observed between acid-suppressive therapy and CDI. Both PPIs and H₂RAs appear to elevate, with the strength of association modified by dose, duration, and concomitant risk factors such as antibiotic use.

**Table 3:** Comparison of articles, study type, and appraisal tool used CDI: *Clostridium difficile* infection; CASP: Critical Appraisal Skills Programme; PPI: proton pump inhibitor; AMSTAR: A MeaSurement Tool to Assess systematic Reviews; SANRA: Scale for the Assessment of Narrative Review Articles

Author	Year	Study type	Appraisal tool	Bias	Purpose
Linsky et al. [11]	2010	Retrospective cohort study	CASP cohort checklist	Moderate	To assess the risk of CDI in hospitalized patients exposed to PPIs and antibiotics.
Mezoff and Cohen [12]	2013	Meta-analysis	AMSTAR	Low	To assess the association between acid suppression and CDI risk.
Finke et al. [15]	2025	Systematic review and dose-response meta-analysis	AMSTAR 2	Low to moderate	To evaluate the dose-dependent risk of CDI with PPI use.
Głaz et al. [16]	2023	Narrative review	SANRA	Moderate	To explore whether PPI-related CDI is a real clinical concern.
Okamoto et al. [17]	2023	Observational single-center study	CASP case series	Moderate	To examine deprescribing rates of antacids after CDI diagnosis.
Huang et al. [18]	2024	Retrospective cohort study	CASP cohort checklist	Moderate	To assess CDI risk based on the duration of PPI or H₂ blocker use.

The studies shown in Table 4 covered a wide range of designs and populations. Large retrospective cohort studies from the United States and Japan included tens of thousands of adults, while single-center observational work involved smaller groups of around 200 patients. Meta-analyses and systematic reviews pooled data from hundreds of thousands of participants across multiple countries, reflecting diverse clinical settings. A narrative review also contributed broader insights by summarizing existing literature without analyzing new patient data. Together, these studies represent both large population-based analyses and focused clinical observations.

**Table 4:** Study designs and populations CDI: *Clostridium difficile* infection; PPI: proton pump inhibitor; H₂RA: histamine-2 receptor antagonist

Author	Study type	Population studied	Setting	Sample size
Linsky et al. (2010) [11]	Retrospective cohort study	Hospitalized adults	US hospitals	>100,000
Mezoff and Cohen (2013) [12]	Meta-analysis of 23 observational studies	Mixed populations	Multicenter/global	>200,000 pooled
Finke et al. (2025) [15]	Systematic review + dose-response meta-analysis	Mixed populations, multiple countries	Multicenter/global	>300,000 pooled
Głaz et al. (2023) [16]	Narrative review	Not applicable (secondary review)	Literature-based	Not applicable
Okamoto et al. (2023) [17]	Observational single-center study	Patients with CDI diagnosis	Japan, single center	~200
Huang et al. (2024) [18]	Retrospective cohort study	Adults on PPIs or H₂RAs	Japan, multicenter	>20,000

One of the strongest and earliest signals was provided by Linsky et al., who demonstrated that hospitalized patients receiving both PPIs and antibiotics had significantly greater odds of developing CDI [11]. More than a decade later, Huang et al. confirmed that risk is strongly associated with treatment duration: patients receiving either PPIs or H₂RAs for more than eight weeks faced significantly elevated CDI risk [18]. This convergence across two large retrospective cohorts underscores that both agent type and exposure duration matter. Table 5 depicts the degree of association between exposure and risk.

**Table 5:** Main findings on association between acid suppression and CDI CDI: *Clostridium difficile* infection; PPI: proton pump inhibitor; H₂RA: histamine-2 receptor antagonist; RCT: randomized controlled trial

Author	Exposure	Main result	Strength of evidence
Linsky et al. (2010) [11]	PPI + antibiotics	Significantly increased CDI risk	Moderate (retrospective)
Mezoff and Cohen (2013) [12]	PPI/H₂RA (varied)	Statistically significant pooled association	Strong (meta-analysis)
Finke et al. (2025) [15]	PPI (cumulative dose)	Dose-dependent risk; higher exposure → greater CDI	Strong (dose-response meta-analysis)
Głaz et al. (2023) [16]	PPI (general review)	Evidence suggestive but confounded; RCTs needed	Moderate (narrative)
Okamoto et al. (2023) [17]	PPI use after CDI diagnosis	High rates of continuation despite CDI	Moderate (observational practice)
Huang et al. (2024) [18]	PPI and H₂RA (duration)	>8 weeks duration associated with elevated risk	Moderate (retrospective cohort)

Meta-analyses further strengthened the evidence base. Mezoff and Cohen pooled 23 observational studies and confirmed a statistically significant association, though they noted heterogeneity in how CDI and exposure were defined [12]. Finke et al. addressed this limitation by adding a dose-response analysis, demonstrating that greater cumulative PPI exposure corresponds with progressively higher CDI risk [15]. The presence of a dose-response gradient satisfies one of Bradford Hill’s criteria for causality and moves the literature closer to causal inference.

Narrative and practice-based studies add nuance to these findings. Głaz et al. critically examined the evidence base and emphasized that although associations are consistent, they remain vulnerable to bias and confounding [16]. Randomized controlled trials, they argued, remain necessary to eliminate residual uncertainty. Okamoto et al. provided a sobering real-world perspective by documenting that many patients continued to receive PPIs even after CDI diagnosis, suggesting a gap between evidence and practice [17].

Table 5 shows that biological plausibility further supports these findings. Gastric acid is a key defense mechanism against ingested pathogens; suppression raises intragastric pH, allowing *C. difficile* spores to survive and reach the intestine. PPIs have also been shown to alter gut microbiota composition and diversity, creating an environment more favorable for pathogenic colonization. When combined with antibiotic exposure, which independently disrupts the commensal flora, the risk of CDI is compounded. Emerging mechanistic work also suggests that PPIs may impair certain immune functions, further compromising host defense.

Despite consistent findings, limitations remain. As Głaz et al. noted, most primary data are derived from retrospective observational designs, which are inherently vulnerable to confounding by indication and misclassification bias. Patients receiving PPIs often have more co-morbidities or greater illness severity, both of which independently predispose to CDI. Moreover, definitions of CDI varied across studies, ranging from laboratory confirmation to administrative coding. Exposure measurement was likewise inconsistent, with many studies relying on prescription data rather than verified patient use. These limitations, while significant, do not entirely undermine the consistency of the evidence. Table 6 outlines the limitations associated with each article.

**Table 6:** Limitations identified across studies CDI: *Clostridium difficile* infection

Author (year)	Key limitations
Linsky et al. (2010) [11]	Retrospective design; confounding by co-morbidities and antibiotics
Mezoff and Cohen (2013) [12]	Heterogeneity in CDI definitions; reliance on observational data
Finke et al. (2025) [15]	Quality of included studies variable; potential residual confounding
Głaz et al. (2023) [16]	No new data; conclusions limited by weaknesses of reviewed studies
Okamoto et al. (2023) [17]	Single-center, small sample; not generalizable
Huang et al. (2024) [18]	Retrospective; possible misclassification of exposure duration

The key limitations across these studies (Table 6) include the retrospective nature of several designs, which introduces confounding factors such as underlying co-morbidities and concurrent antibiotic use, as seen in Linsky et al. [11]. The meta-analysis by Mezoff and Cohen was limited by variability in CDI definitions and dependence on observational data [12]. Finke et al. faced inconsistent study quality and potential residual confounding [15].

From a clinical standpoint, these findings carry several implications. First, PPIs should not be prescribed reflexively or continued indefinitely without indication. In many cases, therapy is initiated for mild or transient symptoms and never reassessed. The evidence reviewed here suggests that this practice carries measurable harm in the form of increased CDI risk. Second, treatment duration must be carefully considered. Where therapy is necessary, shorter courses and lower doses may help mitigate risk. Third, deprescribing should become an integral part of patient management, particularly after CDI diagnosis, as highlighted by Okamoto et al. Integrating PPI stewardship into antimicrobial stewardship programs may provide a systematic mechanism to achieve these goals.

At the policy level, clinical guidelines should more explicitly acknowledge the CDI risk associated with acid suppression. Educational efforts aimed at prescribers could further improve awareness and foster more judicious prescribing practices. Future research should prioritize well-designed prospective studies and, where feasible, randomized controlled trials to more precisely quantify risk. Standardization of CDI definitions and exposure measures is also essential to reduce heterogeneity and improve comparability. Finally, mechanistic studies exploring microbiome disruption and immune modulation may help elucidate the biological underpinnings of this association.

In summary, the evidence strongly supports an association between gastric acid suppression and CDI. The relationship is consistent across study designs, dose- and duration-dependent, and biologically plausible. While observational limitations remain, the cumulative evidence argues for a cautious and judicious approach to PPI prescribing, with emphasis on minimizing unnecessary exposure and implementing deprescribing strategies where appropriate.

## Conclusions

The findings of this review indicate a potential association between gastric acid suppression and the risk of CDI, suggesting it may be an important factor to consider in clinical practice. Several studies have explored this relationship in different patient groups and settings, and emerging mechanistic research has examined how acid suppression might influence the gut microbiome and host defenses. These observations highlight the value of careful prescribing practices, including reviewing the need for ongoing PPI therapy and supporting deprescribing efforts where appropriate. Such approaches may help reduce unnecessary use of acid-suppressive medication and optimize patient care while contributing to overall healthcare quality and safety.
